# Extended reality (XR) in psychosocial and forensic interventions for child and adolescent sexual abuse: a systematic review of current applications and future directions

**DOI:** 10.3389/fpsyg.2025.1687650

**Published:** 2025-11-20

**Authors:** Varinia Leiva, Noemí Pereda, Nicolás Cenzano

**Affiliations:** 1 Faculty of Health and Social Sciences, Universidad de Las Américas, Santiago, Chile; 2 Research Group on Child and Adolescent Victimization (GReVIA), Universitat de Barcelona, Barcelona, Spain; 3 Institute of Neurosciences (UBNeuro), Universitat de Barcelona, Barcelona, Spain; 4 Universidad Adolfo Ibanez Facultad de Ingenieria y Ciencias, Santiago, Chile

**Keywords:** extended reality, child sexual abuse, virtual reality, digital simulation, forensic psychological

## Abstract

The integration of extended reality (XR) technologies—virtual (VR) and augmented (AR)—into child sexual abuse (CSA) interventions has grown over the past decade. This systematic review explores the application, efficacy, and ethical implications of XR tools in psychosocial, forensic, preventive, and therapeutic approaches addressing CSA. Following PRISMA guidelines, a comprehensive search was conducted across Scopus, Web of Science, and PubMed, identifying 11 empirical studies published between 2014 and 2024. Three main intervention categories emerged: (1) professional training, (2) prevention, and (3) therapeutic treatment. Most studies focused on enhancing forensic interviewing skills through avatar-based simulations and immersive training, demonstrating increased use of relevant questions and improved self-efficacy among professionals. Preventive interventions used VR and serious games in school settings to promote body safety awareness and protective behaviors in children. Despite their promise, only one study addressed therapeutic applications directly, highlighting the use of a nonverbal digital tool and serious game to facilitate trauma narration in children with CSA experiences, based on trauma-focused cognitive-behavioral therapy (TF-CBT). The findings emphasize XR's potential to foster engagement, realism, and emotional safety in highly sensitive contexts, specifically in CSA interventions. However, challenges include limited longitudinal evidence, lack of culturally diverse studies, and ethical concerns about exposure, re-victimization, and emotional risks for minors. This review underscores the need for more ethically rigorous research to determine the impact of XR-based interventions in child sexual victimization management.

## Introduction

Child victimization is a serious global issue affecting millions of children, with profound implications for their psychological well-being and development (World Health Organization (WHO), 2022). Victimization includes various forms of violence inflicted upon children and adolescents under 18 years old by adults or peers, occurring in diverse settings such as home, school, and digital environments (Finkelhor, 2008). The experience of violence initiates a probabilistic pathway that triggers a negative developmental cascade (Toth and Manly, 2019), leading to both short- and long-term consequences, including an increased risk of psychopathological symptoms or disorders later in life (Kliethermes et al., 2014).

Child sexual abuse (CSA) is a distinct and particularly severe form of child victimization, defined as any sexual activity involving a child or adolescent where consent is either not obtained or inherently impossible (Johnson, 2004). This includes both physical acts (e.g., inappropriate touching, intercourse, rape) and non-physical acts (e.g., exposing a child to sexual content or behaviors). CSA is fundamentally rooted in the abuse of power and the betrayal of a child's trust, often resulting in severe psychosocial harm (Hinds and Giardino, 2020). Prevalence estimates suggest that CSA affects a significant proportion of children worldwide, necessitating urgent and effective intervention strategies (Piolanti et al., 2025).

### The role of technology in CSA interventions

While digital technologies have been associated with increased risks of cybervictimization among children and adolescents (Strohmeier and Gradinger, 2022), they also present opportunities for innovative intervention strategies (Nocentini et al., 2015). Digital tools have been successfully integrated into interventions for CSA victims, with applications spanning prevention, therapeutic treatment, and forensic assessment (Rowe et al., 2014; Sánchez-Jiménez et al., 2024).

The advancement of digital technology has led to the rapid expansion of Extended Reality (XR), an umbrella term encompassing Virtual Reality (VR) and Augmented Reality (AR). XR technologies extend reality through digital means, providing novel, immersive, and interactive experiences for users (Rauschnabel et al., 2022). The emergence of XR technologies provides a promising avenue for enhancing interventions within both psychosocial and forensic contexts (Baugerud et al., 2024; Segal et al., 2024).

VR fully immerses users in simulated digital environments, which can be experienced through large projection screens or head-mounted display (HMD) systems. The effectiveness of VR is closely tied to immersion and telepresence, enabling users to feel as if they are present within the virtual environment rather than in their physical surroundings (Dalton, 2021; Freeman et al., 2017). In contrast, AR overlays digital visual information onto real-world environments in real-time, presented via smartphones, tablets, smart glasses or other gadgets. The effectiveness of AR is often measured by realism—the extent to which users perceive the hybrid environment as authentic (Hilty et al., 2020).

VR has gained considerable attention in clinical psychology and mental health research as an effective tool for therapeutic interventions (Riva et al., 2019). Given that mental health disorders are closely intertwined with environmental factors (Leiva et al., 2021), VR's ability to create controlled, immersive environments allows for evidence-based interventions that support patients in confronting distressing experiences (Freeman et al., 2017). One of the most widely used XR-based interventions in clinical psychology is Exposure Therapy, where individuals are gradually exposed to anxiety-inducing stimuli within a controlled virtual setting (Botella et al., 2017). Although AR-based interventions remain relatively underexplored compared to VR, AR holds unique advantages, including greater affordability, ease of stimulus development, and the ability to integrate therapeutic content into real-world settings (Vinci et al., 2020).

#### XR in psychosocial interventions for CSA

Within the domain of child victimization, XR technologies offer significant potential for improving psychosocial interventions. The ability of XR to create safe, controlled, and immersive environments enables innovative therapeutic approaches, particularly in trauma-focused interventions. Serious games (SG), which integrate gaming mechanics for educational or therapeutic purposes, have also been applied in child maltreatment prevention and treatment. Studies focusing on CSA indicate that VR and AR, when combined with SG, can be effective in engaging children in intervention programs.

Emerging evidence supports the potential of VR as a promising tool in the psychosocial treatment of sexual violence. For instance, the intervention *Sister, I will tell you!* integrated holographic storytelling with reflective writing and mindfulness meditation over a 4-week period. Compared to an audio-guided control group, participants in the VR condition reported greater perceived social support, significant reductions in the long-term psychological impact of sexual violence and decreased suicidal ideation. Although conducted in adults and focused on early recovery, the study highlights how VR-based, self-directed interventions—especially those that reduce barriers related to stigma and disclosure—can play a meaningful role in supporting survivors of sexual trauma (Lee and Cha, 2020).

However, further research is needed to determine the most effective XR-based applications for specific intervention goals. Recent studies have explored the use of VR in managing psychological distress among adolescents, demonstrating its potential to reduce anxiety and improve coping mechanisms (Neumann et al., 2021). Additionally, VR-based interventions have been developed to support imaginal exposure within PTSD treatment, offering immersive environments that facilitate therapeutic processes (Kip et al., 2023).

#### XR in forensic interventions for CSA

Beyond psychosocial applications, XR technologies are increasingly being explored in forensic contexts related to CSA cases. XR environments have been proposed as tools for conducting child-friendly forensic interviews, aiming to reduce the stress and trauma associated with traditional legal procedures (Baugerud et al., 2024; Kip et al., 2024), as well as to support the assessment of perpetrators (Renaud et al., 2014). Additionally, XR has emerged as a promising tool in criminology, enabling the simulation of specific scenarios in controlled, safe, and ecologically valid environments for the analysis of criminal behaviors. This ability to recreate complex and realistic interactions makes XR particularly valuable for understanding and evaluating forensic phenomena (Van Gelder, 2023).

Exploratory studies further suggest that VR may enhance forensic assessment in suspected CSA cases, especially for identifying individuals with deviant sexual arousal patterns. When combined with physiological measures such as penile plethysmography, VR has demonstrated greater accuracy in distinguishing sexual responses between individuals with a history of child sexual victimization offenses and non-deviant profiles (Marschall-Lévesque et al., 2017; Renaud et al., 2014).

Taken together, these findings highlight VR's growing relevance as a complementary and innovative approach for improving the sensitivity, accuracy, and ecological validity of forensic evaluations in both victims and perpetrators of CSA.

### Aim of the study

The integration of XR technologies into psychosocial and forensic interventions for CSA represents an emerging field with significant implications for research, practice, and policy. Several studies have been published on the use of XR technologies with sexual perpetrators (i.e., Schröder et al., 2023). However, no previous systematic review has comprehensively examined the state of the art regarding XR-based interventions with child and adolescent victims. This systematic review aims to explore the current applications of XR technologies in psychosocial and forensic responses to CSA, evaluating their effectiveness, challenges, and future directions. By synthesizing existing evidence and identifying critical gaps, this review seeks to inform researchers, practitioners, and policymakers on the potential of XR interventions to enhance prevention, assessment, and therapeutic support in cases of CSA.

## Method

The selection process for the studies included in this systematic review followed the Preferred Reporting Items for Systematic Reviews and Meta-Analyses (PRISMA) (Page et al., 2021).

## Search strategy

A comprehensive search was conducted using the databases Web of Science, Scopus, and PubMed US, with the latest search conducted between 2014 and 2024. The search configuration included (“Child^*^” OR “Adolesc^*^”) AND “Victim^*^” AND (“Sexual violence” OR “Sexual abuse”) AND (“Digital techniques“ OR ”Extended reality“ OR ”Virtual reality“ OR ”Augmented reality“). No temporal or sample size limits were set, with the aim of including all publications on the topic. Duplicated articles were identified and removed from the list of documents found, ensuring the integrity of the dataset.

### Eligibility criteria

This systematic review included empirical studies published between 2014 and 2024 in English or Spanish that examined the application of XR technologies, including VR, AR, and SG, in the psychological, social, or forensic intervention of CSA. Eligible studies focused on interventions aimed at prevention, assessment, or treatment, encompassing both direct interventions with children and adolescents (0–18 years old) who have experienced sexual abuse and interventions targeting professionals involved in CSA cases, such as forensic interviewers, mental health practitioners, and legal professionals (Baugerud et al., 2024; Segal et al., 2024).

Both quantitative and qualitative studies were included, provided they employed a clearly defined empirical methodology with rigorous data collection and analysis procedures. Qualitative studies were required to use structured methodologies, including semi-structured interviews, thematic analysis, or ethnographic approaches, ensuring empirical validity (Sánchez-Jiménez et al., 2024). Studies were excluded if they were non-empirical, lacked a clearly described methodology, did not focus on interventions related to CSA, or did not incorporate XR-based applications in their design.

**Figure 1:**
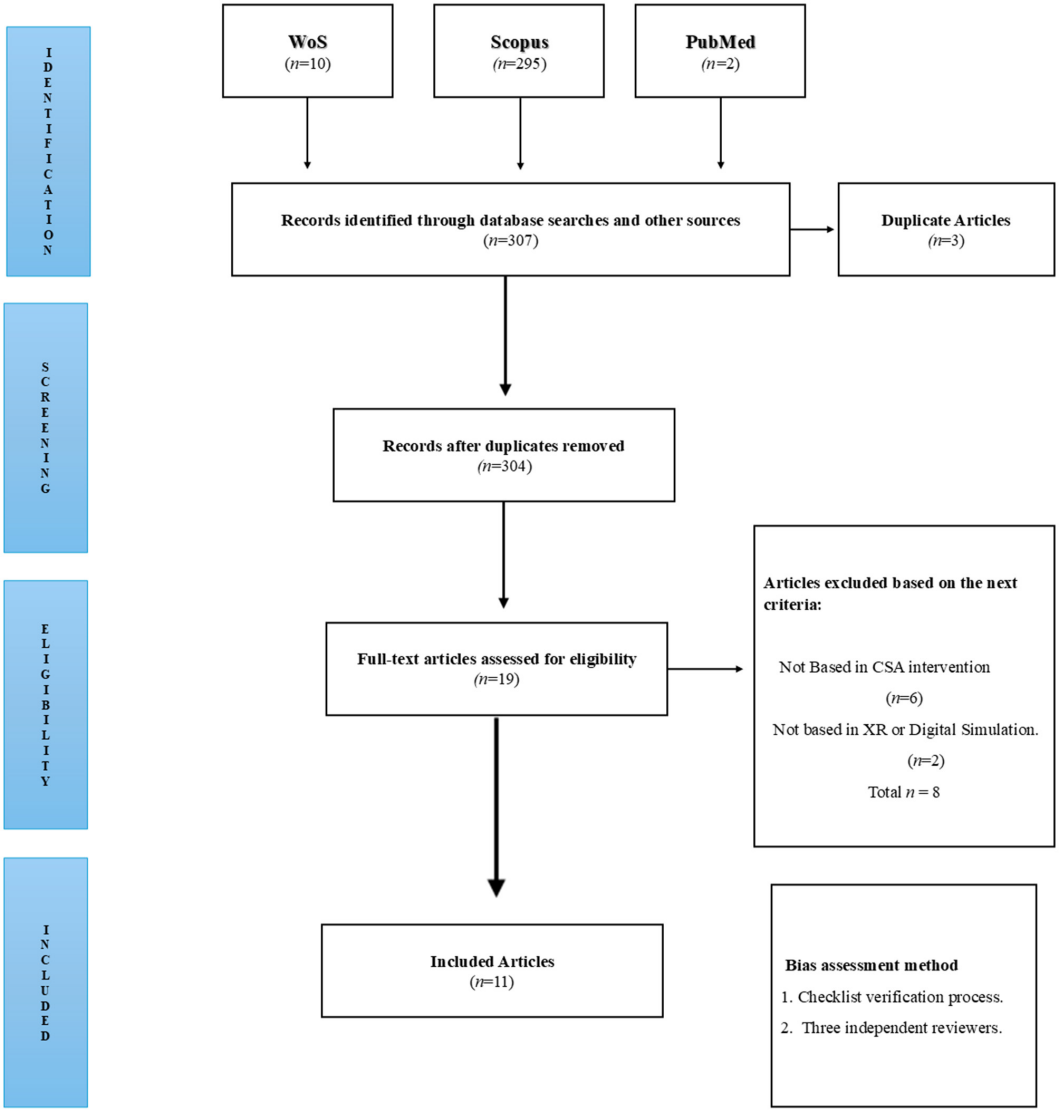
PRISMA flow diagram summarizing the study selection process.

The flow diagram in Figure 1 presents the detailed process of study selection.

A total of 307 records were initially identified through database searches in Web of Science, Scopus, and PubMed. After removing three duplicates, 304 titles and abstracts were screened for relevance. Of these, 285 records were excluded for not meeting the inclusion criteria. The full texts of the remaining 19 articles were then assessed for eligibility. Following a detailed review, 8 articles were excluded for reasons such as not meeting population criteria, lacking relevant outcome measures, or employing inadequate methodology. Ultimately, 11 studies were included in the final synthesis.

### Data coding

Several characteristics of the studies were coded in relation to 13 differentiated aspects. First, regarding bibliometric indicators, we coded (a) name of signatory authors and/or organizations, (b) year of publication, (c) their country of origin and (d) the main results. Besides, we coded several methodological aspects of the studies such as (e) method, (f) geographic location of study, (g) object of study, (h) study participants, (i) sample size, (j) participant age range, (k) participant racial/ethnic/national background, (l) program's characteristics, and (m) technology used.

### Quality assessment

The quality of included studies was assessed using established PRISMA checklists. Additionally, to ensure objectivity and consistency, the three authors independently analyzed the search results. In addition to the overall PRISMA-based quality analysis, an individualized risk of bias assessment was conducted for each included study. This evaluation considered methodological design, sample size, participant characteristics, and transparency in data reporting. Each study was independently reviewed by the three authors, and any discrepancies were resolved by discussion until consensus was reached. When a study presented notable limitations or potential bias, these were explicitly reported in its specific results and highlighted in the discussion and limitations section of this review.

## Results

The systematic review identified 11 studies exploring the application of XR and digital simulation technologies in the context of CSA. These studies were categorized into three main areas: professional training, prevention, and therapeutic interventions. No studies were found that involved direct assessment with children who had been victimized. However, some addressed professional training focused on assessment and investigative procedures involving victimized children.

As shown in Table 1, a significant proportion of the studies (63.6%) focused on training professionals, employing technologies such as VR and digital simulations to enhance the competencies of practitioners including psychologists, social workers, and law enforcement officers. These interventions are designed to strengthen professionals' abilities to recognize, respond to, and manage cases of CSA effectively. Additionally, three studies (27.2%) concentrated on prevention strategies, using VR and SG to educate children and adolescents about CSA. These initiatives aim to increase awareness, promote protective behaviors, and empower young people to identify and report inappropriate or high-risk situations. Only one study addressed therapeutic interventions, underscoring the early stage of integrating XR technologies into clinical treatment for CSA survivors. This study explored the use of SG to support trauma processing and emotional expression within therapeutic contexts.

**Table 1:** Studies employing XR and digital simulations in CSA.

**Study**	**Topic**	**Sample origin**	**Technology**
Pompedda et al. (2020)	Training professionals	Italy and Estonia	Virtual reality. Simulated avatars responding based on probabilistic algorithms replicating real children's responses
Haginoya et al. (2020)	Training professionals	Japan	Culturally adapted web-based avatar simulation where participants interviewed virtual children and received automated feedback based on question type, with video responses triggered by a coded algorithm
Kask et al. (2022)	Training professionals	Estonia	Digital simulation (avatars)
Hassan et al. (2023)	Training professionals	Norway	Virtual reality, two-dimensional (2D) desktop, interactive audio, text chat
Røed et al. (2023)	Training professionals	Australia, United Kingdom and Singapore	Digital simulation
Krause et al. (2024)	Training professionals	Germany	Virtual reality, natural language processing, adaptive feedback system with AI-based evaluation, online seminar training platform
Segal et al. (2024)	Training professionals	Lithuania	Digital simulation (avatars)
Rowe et al. (2014)	Prevention	United States	Virtual reality
Stieler-Hunt et al. (2014)	Prevention	Australia, United Kingdom and Singapore	Serious gaming with interactive virtual scenarios
Sánchez-Jiménez et al. (2024)	Prevention	Spain	Virtual reality
Endendijk et al. (2021)	Therapeutic intervention	Netherlands	Serious gaming with interactive scenarios

### XR-based training for professionals interviewing CSA victims

This type of intervention emerged as the most frequently studied application of XR technologies in the field of child sexual victimization. An increasing body of empirical research supports the integration of XR into professional training programs for conducting investigative interviews with CSA victims. These tools—including VR, 2D desktop simulations, and multimodal platforms—simulate interviews with child avatars to enhance interviewers' practical skills in realistic, emotionally charged scenarios. The studies reviewed involved diverse participant profiles, including university students, educators, child protection workers, psychologists, and law enforcement professionals.

Seven studies specifically examined the use of XR tools—VR, 2D simulations, and AI-driven avatars—for training professionals in interviewing children exposed to CSA. These interventions consistently demonstrated improvements in adherence to best practice interviewing guidelines, notably through an increased use of open-ended questions, enhanced empathetic engagement, and a reduction in suggestive or leading prompts.

A common feature across these studies was the use of interactive child avatars, programmed using probabilistic algorithms and machine learning models informed by authentic child response patterns. These avatars adjusted their responses based on the types of questions posed, allowing participants to experience emotionally evocative, developmentally appropriate interview scenarios with child avatars typically representing school-aged children. This dynamic interaction fostered the acquisition of essential competencies for investigative interviewing. Table 2 outlines the methodological details of these seven studies.

**Table 2:** Characteristics of studies on XR-based training for investigative interviews in CSA cases.

**Authors**	**Program**	**Sample**	**Method**	**Tools**
Haginoya et al. (2020)	Not specified	The participants were 32 (23 women) university undergraduates (*M =* 20.5 years old, SD = 0.6). They were randomly allocated into either the feedback (*n =* 17) or the control group (*n =* 15).	Randomized controlled trial.	**Question-Type Coding System** based on the *NICHD Investigative Interview Protocol*. **Algorithmic scoring** of *relevant, neutral, and incorrect details* according to predefined probabilities in the avatar database. **Reliable Change Index (RCI)** to detect statistically significant improvement in the proportion of recommended questions between first and last sessions
Pompedda et al. (2020)	The Empowering Interviewer Training (EIT)	Study I involved 40 licensed psychologists in Italy (*M =* 27 years old); Study II included 69 psychology students in Estonia (*M =* 23 years old).	Randomized controlled trial comprising two studies. Participants were randomly assigned to either a control group (no feedback) or an experimental group (feedback provided).	**Interview Quality Coding Manual** derived from the *NICHD Protocol* (recommended vs. non-recommended question types). **Accuracy Coding Scheme** for *correct vs. incorrect details* elicited from avatars and real children. **Rapport Building and Interview Quality Checklist** adapted from *NICHD Guidelines*. **Multilevel modeling** for transfer of training effects into real interviews
Kask et al. (2022)	The Empowering Interviewer Training (EIT)	17 police investigators (2 men, 15 women), with an average age of 40.5 years. They were randomly allocated into either the feedback group (*n =* 11) or the no feedback group (*n =* 9).	Feedback Group: Officers participated in one avatar training session, where they conducted four interviews with avatars and received immediate feedback after each session. They provided two real child interview transcripts, one before and one after training. No Feedback: Officers first conducted four avatar interviews without feedback.	**EIT Coding Scheme:** classification of question types (invitations, facilitators, directives, option-posing, suggestive). **Accuracy scoring** of details recalled by children. **Inter-rater reliability** using Cohen's κ to ensure coding consistency.
Hassan et al. (2023)	Not specified	40 child protection professionals and child welfare students.	Randomized controlled trial. Mixed design.	**Quality of Experience Questionnaire (ITU-T P.809)** assessing five QoE metrics: *realism, presence, responsiveness, flow*, and *overall quality*. **Flow State Scale (FSS)** and *Engagement in Learning* subscale for motivation. **Self-Efficacy Questionnaire** for perceived learning impact. **Automated Question Classification Model** (open-ended vs. closed-ended; *balanced accuracy = 0.87*) for objective performance scoring
Røed et al. (2023)	LiveSim Training Platform	The study analyzed real-world data from 606 professionals, including child protection workers, police officers, and psychology/social work trainees, collected over a 9-year period (2009–2018) across Australia, the United Kingdom, and Singapore.	For the descriptive analysis, engagement and internal features of LiveSim were evaluated using Visual Basic for Applications in Excel. Additionally, a compatibility assessment was conducted to test the program's access and functionality across different hardware systems.	**System-embedded analytics** of engagement and task performance (interaction logs, completion rates, feedback frequency) processed via *Visual Basic for Applications in Excel*; **usability and compatibility assessments** across hardware setups following *ISO 9241-210 usability criteria*.
Krause et al. (2024)	ViContact	110 student teachers (92 women, 17 men) from Psychologische Hochschule Berlin, Germany, were randomly assigned to one of four groups: VR training (*n =* 27), seminar training (*n =* 26), combined VR and seminar training (ST + VR, *n =* 29), or a control group (*n =* 28).	Randomized-controlled trial.	**Interview-quality coding** using the *Empowering Interviewer Training/NICHD scheme* for question types and detail accuracy; **Post-Session Self-Efficacy Scale** (adapted from the Motivated Strategies for Learning Questionnaire, MSLQ); **Presence Questionnaire (PQ)** for immersion and engagement.
Segal et al. (2024)	Avatar simulation system to examine the emotional responses	30 psychology students and recent graduates (22 female, 8 male, *M =* 27.9 years old, SD = 7.15).	Within-subject design to examine participants' emotional reactions to child avatars disclosing CSA vs. non-CSA scenarios.	**Self-Assessment Manikin (SAM)** for valence and arousal; **Interpersonal Reactivity Index (IRI)** subscales (*Empathic Concern, Personal Distress*); **Behavioral observation** of facial affect and engagement during avatar interaction.

Haginoya et al. (2020) assessed the effectiveness of a digital simulation training program featuring child avatars to improve investigative interviewing skills in CSA contexts, adapted from a Western to a Japanese cultural setting. The program consisted of six simulated interviews with avatars representing 4–6 year-old children exposed to abuse or non-abuse scenarios. Avatars responded probabilistically, based on empirical data on children's disclosure patterns. University students were randomly assigned to a feedback or control group, with only the former receiving structured, immediate feedback on their questioning style after each session. The results showed that participants in the feedback group significantly improved their interview quality from the fourth session onwards, increasing their proportion of recommended questions from 31.4% to 56.3%. Additionally, they elicited more correct and fewer incorrect details compared to the control group in the post-training assessment. The authors concluded that avatar-based simulation with feedback is an effective, scalable strategy for strengthening evidence-based interviewing skills, even among novice participants, though at least four training sessions may be necessary to achieve significant improvements.

Pompedda et al. (2020) developed an interactive simulation platform aimed at enhancing investigative interviewing skills through immersive, feedback-based training. The platform featured 16 child avatars aged 4–6, programmed with probabilistic algorithms to encourage the use of recommended open-ended questions. Participants, including psychologists and psychology students, conducted simulated interviews and received detailed feedback on their techniques. The results indicated that those receiving avatar-based training with individualized feedback increased their use of recommended open-ended questions by 61% and elicited significantly more correct details (*p* < 0.001). Notably, psychologists demonstrated a significant transfer of skills to real child interviews, while students' improvements were less consistent. The authors emphasized the potential of AI-driven SG training tools to bridge the gap between theoretical knowledge and applied practice.

Kask et al. (2022) extended these findings to law enforcement professionals, reporting that participants receiving structured feedback during avatar simulations increased their use of recommended questions by 58% and decreased their use of suggestive prompts by 71% relative to controls (*p* < 0.001). Additionally, child responses during these interviews were more comprehensive and intelligible. The study highlighted the value of integrating structured, feedback-based XR training for law enforcement professionals and recommended further research on sustaining long-term skill retention.

Krause et al. (2024) evaluated a VR-based training program designed for student teachers conducting sensitive conversations about suspected CSA. The program included a 2-h VR session with virtual child avatars and automated, personalized feedback, complemented by a 2-day online seminar covering CSA knowledge, legal frameworks, and interview techniques. The combined training group (seminar + VR) significantly increased their use of recommended questions (from 41% to 84%; *p* < 0.001) and improved self-efficacy scores, while minimal gains were observed in groups receiving only one training format. The authors concluded that VR should complement rather than replace traditional training, underscoring the value of multimodal pedagogical approaches.

Hassan et al. (2023) compared four training formats—VR, 2D desktop simulation, audio-only, and text-based scenarios—among child protection workers and students. Participants engaged in brief sessions with a virtual child avatar, with avatar responses generated by a dialog model trained on mock interview transcripts. While no significant differences were found in question quality across formats, VR was consistently rated as the most immersive and engaging, particularly by professionals. Additionally, an automated feedback tool utilizing machine learning achieved 87% accuracy in classifying question types, highlighting opportunities for scalable, adaptive training solutions. The authors concluded that VR offers the most effective training medium due to its immersive and realistic properties.

Røed et al. (2023) reported on the large-scale implementation of LiveSim, a cloud-based, avatar-driven training platform for investigative interviewing. Featuring a virtual 5-year-old child avatar and a decision tree framework, the platform reinforced the use of open-ended and non-leading questions. Data from 606 professionals across over 11,000 sessions demonstrated sustained engagement and progressive improvement in recommended question use. Although inferential statistics were not reported, descriptive data strongly supported the program's feasibility, replicability, and cost-effectiveness, positioning LiveSim as a promising alternative to traditional role-play training with actors.

Segal et al. (2024) explored participants' emotional responses during avatar-based simulations involving CSA disclosures versus neutral narratives. Using child avatars disclosing nine sequential details—six suggesting CSA and three confirming or disconfirming victimization—psychology students' emotional reactions were measured via facial expression analysis software. The study controlled for individual differences in baseline emotionality and perceived avatar realism. The results indicated stronger negative emotions (anger, sadness, disgust) in response to confirmed CSA scenarios (*p* < 0.001), with participants who perceived avatars as more realistic or reported higher emotionality showing heightened reactions. The authors emphasized the importance of balancing ecological validity with emotional safety in XR-based training environments, advocating for emotional support mechanisms to prevent affective overload.

Across the included studies, the assessment of professional competencies and emotional outcomes (e.g., empathy, self-efficacy, engagement) was based on standardized self-report questionnaires, quality-of-experience measures, and interview coding schemes, depending on each study's design. The specific instruments used in each case are summarized in Table 2.

Regarding to transfer of learning to real child interviews was evaluated in two studies. Pompedda et al. (2020) assessed interviewers' performance one week after avatar training by coding real interviews with children who had witnessed a mock event, using the NICHD protocol to quantify recommended questions and accuracy of elicited details. Similarly, Kask et al. (2022) analyzed pre- and post-training transcripts of actual investigative child interviews coded with the Empowering Interviewer Training (EIT) scheme. The remaining studies examined performance within simulated environments only.

### XR-based interventions for the prevention of child sexual abuse

Three studies investigated the use of XR and SG in the prevention of CSA, targeting various age groups during childhood and utilizing different technological formats. These interventions showed promising results in enhancing knowledge, shaping attitudes, and promoting protective behaviors among children. However, challenges related to scalability, cultural adaptation, and integration into existing prevention frameworks persist. Table 3 summarizes the methodological characteristics of these studies.

**Table 3:** Characteristics of Studies on XR-Based Interventions for CSA Prevention.

**Authors**	**Program**	**Sample**	**Method**	**Tools**
Rowe et al. (2014)	My Voice, My Choice	83 adolescent girls aged 14–18 (*M =* 15.63 years old, SD = 0.95) from an urban public high school, randomized to the MVMC intervention (*n =* 47) or a wait-list control group (*n =* 36).	Randomized controlled pilot trial.	**Conflict in Adolescent Dating Relationships Inventory (CADRI):** 25-item measure assessing sexual, physical, and psychological victimization by male peers. **Trauma Symptom Checklist (TSC):** Measures psychological distress (PTSD, depression, anger control). **Participant Satisfaction and Engagement Questionnaire:** Likert-scale assessing program enjoyment, involvement, and perceived usefulness (researcher-developed). **Facilitator/Actor Engagement Ratings and Fidelity Checklist:** Observational ratings of participant engagement and adherence to protocol.
Stieler-Hunt et al. (2014)	Orbit	Subject matter experts and small pilot groups of 8–10-year-old children for playtesting; no formal sample size reported.	Case study of the design and development of a serious game aimed at CSA prevention for schoolchildren aged 8 to 10 years old.	**Children's Knowledge of Abuse Questionnaire (CKAQ):** assesses children's understanding of key sexual abuse prevention concepts (e.g., body boundaries, appropriate/inappropriate touch). **What-If Situation Test (WIST):** measures children's ability to identify unsafe or abusive situations and indicate appropriate protective responses (“need to tell” vs. “do not need to tell”). **Game analytics and observation logs:** used during pilot play-testing to evaluate usability, engagement, and emotional responses. **Teacher feedback forms and classroom observation checklists:** designed to assess integration of the game with school activities and children's reflective discussions. **Pre–post evaluation with control group:** planned to measure knowledge and behavioural-intention changes after game exposure, comparing results against non-intervention classrooms.
Sánchez-Jiménez et al. (2024)	Virtual-PRO	579 Spanish adolescent students aged between 12 and 17 years (*M =* 14.76 years old, SD = 0.88; 47.1% boys) were randomly grouped into experimental (*n =* 286) and control (*n =* 293) conditions.	Randomized-controlled trial.	**Inventory of Ambivalent Sexism in Adolescents (ISA)**. **Moral Disengagement Scale** **Bystander Intention to Intervene in Sexual Harassment**; three written scenarios (physical/online/verbal harassment); response options (e.g., do nothing, walk away, join in, tell aggressor to stop, get help from others). **Sexual Harassment Survey—Spanish adaptation/validation**-−20 items; **Victimization** and **Perpetration**. **Peer Sexual Cybervictimization Scale-** **VR experience checks (study-specific single-item questions)**—**Realism**, **Emotional Impact**, and **Embodiment, (**administered immediately after the VR scenarios within the app. **Program adherence** and **satisfaction**.

Rowe et al. (2014) developed a single-session, 90-min intervention aimed at reducing sexual victimization among adolescent girls by enhancing their assertive resistance skills. Delivered within an immersive virtual environment (IVE), the program simulated coercive real-life situations, such as peer pressure to engage in physical intimacy. Through guided role-play within the IVE, participants practiced resisting unwanted advances and received immediate feedback on both their verbal and non-verbal responses. The immersive setting was designed to replicate the emotional and cognitive demands of high-risk situations, thereby promoting skill acquisition and emotional readiness in a safe, controlled context. The study found that My Voice, the intervention program, significantly reduced CSA incidents among adolescent girls over a 3-month follow-up period. Only 10% of participants in the experimental group reported experiencing sexual victimization, compared to 22% in the control group (OR = 0.47, *p* < 0.05), representing a 35.4% risk reduction. Moreover, the intervention was particularly beneficial for girls with a history of victimization, who experienced lower levels of psychological victimization (*b* = −0.19, *p* < 0.01) and reduced psychological distress (*b* = −0.55, p =0.05) over time. Symptoms of psychological distress, including PTSD-related stress, depression, and anger control difficulties, also showed significant reductions among participants with prior victimization. While the intervention had no significant effect on physical victimization rates, it effectively improved assertive resistance skills and increased participants' confidence in responding to coercive situations.

Virtual-PRO, a school-based, curriculum-integrated program developed in Spain, utilized immersive VR to address peer sexual harassment among adolescents aged 12–17 (Sánchez-Jiménez et al., 2024). Delivered over six sessions, the program combined VR simulations, role-playing exercises, and classroom discussions. The intervention significantly reduced moral disengagement, visual/verbal and online victimization, and mitigated increases in both hostile and benevolent sexism. It also improved participants' intention to intervene as bystanders, particularly when the victim was not a peer. These effects were maintained at a 3-month follow-up, supporting the potential of VR-enhanced psychoeducational programs to influence cognitive, moral, and behavioral dimensions related to sexual violence. However, reductions in sexual aggression were not observed, and the authors noted that the high implementation costs associated with VR equipment posed challenges for broader dissemination.

Stieler-Hunt et al. (2014) developed Orbit, a SG designed as a digital prevention intervention for children aged 8 to 10. The program integrated a narrative-based adventure game with mini-games, avatar customization, and classroom activities. Key design elements included emphasizing the role of trusted adults, addressing barriers to disclosure, and depicting realistic abuse scenarios without excessive sanitization. The game supported children's understanding of body safety, fostered a positive self-concept, and promoted emotional engagement. Additionally, Orbit involved adults—parents and teachers—through parallel activities and login features. The program represents an innovative, scalable approach to CSA prevention, utilizing interactive storytelling, SG mechanics, and trusted adult involvement. By balancing engagement, realism, and evidence-based design, the authors conclude that Orbit offers a promising tool for early education on personal safety and abuse prevention. While developed with strong theoretical and expert-informed foundations, the effectiveness of Orbit has not yet been empirically validated.

### XR-based interventions for CSA treatment

Although interest in XR technologies for the prevention of and professional training around CSA has increased in recent years, few studies have examined their use in psychotherapeutic treatment specifically targeting CSA. The characteristics of these interventions are summarized in Table 4.

**Table 4:** Characteristics of studies on XR-based interventions for CSA treatment.

**Authors**	**Program**	**Sample**	**Method**	**Tools**
Endendijk et al. (2021)	Vil Du?!	20 therapists using Vil Du?! were invited to complete an online questionnaire after each use with a client. 23 children with CSA (*M =* 11.38 years old, SD = 3.96, Mi*n =* 5, Max = 18; 61% female). Twelve therapists volunteered for interviews, and 10 participated. Total number of therapists in the survey phase remains unknown due to anonymous responses.	A mixed-methods triangulation design.	Custom **online questionnaire** (LimeSurvey) collecting therapists' reports on when, how, and why *Vil Du?!* was used across CBT components (psychoeducation, trauma processing, self-protection). **Semi-structured interviews** explored therapists' experiences and perceived client responses; data analyzed with **NVivo** and descriptive statistics (SPSS). **Limitation:** No standardized quantitative outcome measures were used; results rely solely on therapist self-reports.

The study by Endendijk et al. (2021) represents a pioneering effort in evaluating the integration of a SG, Vil Du?!, within trauma-focused cognitive behavioral therapy (TF-CBT) for children and adolescents who have experienced CSA. Data were collected from 23 therapy cases and 10 in-depth interviews with therapists. Findings indicated that the game was most commonly used to support trauma narration and processing, followed by psychoeducation on sexuality and, to a lesser extent, the development of self-protection skills. Vil Du?! employs a non-verbal, avatar-based interface that enables children to symbolize abusive experiences through interactive, synchronized actions between avatars, providing an alternative communication channel for those facing verbal, emotional, or cognitive barriers to disclosure.

Therapists emphasized the game's value for clients experiencing shame, avoidance, or anxiety, highlighting its ability to facilitate gradual exposure to trauma-related content within a controlled, child-led framework. However, some concerns were raised regarding the risk of suggestive questioning and the necessity of structured therapist training to ensure ethical and effective use. While preliminary and not designed to assess clinical outcomes, the study offers important insights into the therapeutic potential of XR-based serious games and underscores the need for implementation protocols and longitudinal research to evaluate their clinical efficacy in CSA interventions.

## Discussion

Over the past decade, the integration of technology into psychological interventions has expanded markedly, incorporating XR, online platforms, and, more recently, AI-based tools (Cruz-González et al., 2025; Riva and Serino, 2020). Despite this growth, their application in interventions involving children—particularly in sensitive and high-risk contexts such as child victimization—remains limited. In the specific field of CSA, this systematic review identifies a growing, yet still exploratory, interest in the use of XR technologies between 2014 and 2024. Notably, while VR and SG have begun to gain visibility, the application of AR in CSA-related interventions remains largely absent from current research.

The findings of this review point to a rising global interest in the potential of XR technologies to address CSA across diverse cultural and institutional settings, with a marked predominance of research produced in European countries. Moreover, it is possible to distinguish three primary domains in which XR has been applied within CSA intervention frameworks: (1) professional training for practitioners working with victims, (2) prevention initiatives aimed at children and adolescents, and (3) therapeutic treatment, the latter of which remains in a nascent and largely exploratory phase.

Regarding professional training, XR—primarily through VR and avatar-based simulations—has been employed to replicate investigative interviews with victimized children. These tools are predominantly applied within forensic and investigative contexts, aiming to enhance professionals' adherence to evidence-based guidelines for interviewing and assessing children who have experienced CSA. The available evidence suggests that XR-based simulations, particularly when integrated with structured feedback and embedded within comprehensive pedagogical frameworks, improve practitioners' ability to formulate open-ended, non-suggestive questions while also fostering emotional readiness to manage complex and emotionally charged CSA disclosures. Positive outcomes have been observed across a wide range of professional profiles, including psychologists, social workers, law enforcement personnel, teacher trainees, and university students. Notably, several studies reported transfer effects from virtual training environments to real-life interview contexts, reinforcing the ecological validity and practical utility of XR-based training methodologies (Kask et al., 2022; Krause et al., 2024; Stieler-Hunt et al., 2014).

Moreover, immersive VR environments were consistently rated as highly engaging and emotionally impactful, enhancing participants' sense of presence and perceived learning. These findings align with previous literature emphasizing the importance of immersive realism for maximizing training outcomes (Bowman et al., 2020; Dalton, 2021; Freeman et al., 2017). However, the effectiveness of these tools appears to vary according to participants' baseline expertise, cognitive load, and training context—an observation consistent with prior research (Gu et al., 2022; Pompedda et al., 2020). Emotional engagement emerged as a particularly important factor influencing training efficacy: while realistic avatars elicited strong affective responses that can enhance memory consolidation, they also highlighted the need to integrate emotional regulation strategies within training programs to mitigate potential distress (Ligthart et al., 2021; Seinfeld et al., 2018).

Although avatar-based training programs have demonstrated scalability and cost-effectiveness, further research is required to refine AI-driven feedback systems, improve avatar responsiveness to spontaneous verbal input, and assess the sustainability of training effects over time. Overall, XR-based professional training in CSA contexts appears to be an ethically sound and promising intervention. These tools consistently improve adherence to investigative interviewing protocols, increase the use of open-ended questions, enhance the quality of elicited information, and bolster professionals' self-efficacy and emotional preparedness.

Nevertheless, a recurrent limitation across studies is the scarcity of long-term follow-up data, limiting the ability to evaluate the durability of training effects over time. Future research should prioritize longitudinal designs, investigate potential differential effects across professional groups, and explore the integration of adaptive, AI-enhanced feedback systems with human feedback and supervision. Furthermore, attention to emotional safety and cultural contextualization within XR simulations remains essential to ensure both the effectiveness and ethical integrity of training programs designed for professionals working with CSA victims.

In the domain of prevention, XR applications—primarily through immersive VR experiences and SG—have demonstrated considerable potential as psychoeducational tools for developing self-protection skills among children and adolescents. These programs are predominantly designed for school-based implementation, where their interactive and experiential nature aligns well with educational objectives. For younger children, SG interventions have proven especially effective due to their narrative-driven, interactive formats, which foster engagement and support learning processes—findings consistent with prior literature on digital learning tools for children (Baranowski et al., 2015; Boboc and Damaševičius, 2024). Within this review, the Orbit program identified several essential design components associated with preventive efficacy in child sexual victimization: the creation of engaging digital environments, the active involvement of trusted adults to facilitate real-life applicability, and the deliberate avoidance of over-sanitized content to help children grasp the nuanced, sometimes uncomfortable realities of abuse (Stieler-Hunt et al., 2014). For adolescents, prevention strategies shifted toward enhancing conceptual understanding of CSA, recognition of risk scenarios, and the development of assertive resistance skills. These programs employed immersive role-playing experiences to simulate realistic coercive situations, enabling participants to rehearse protective responses within a controlled, emotionally engaging context (Rowe et al., 2014; Sánchez-Jiménez et al., 2024). Importantly, all prevention interventions reviewed were grounded in empirically supported or theoretically informed CSA prevention frameworks, reinforcing their validity and highlighting the critical importance of adapting technological interfaces to complement evidence-based content (Bowman et al., 2020; Riva and Serino, 2020; Sánchez-Jiménez et al., 2024; Stieler-Hunt et al., 2014). In line with previous findings, several studies also emphasized the value of integrating digital interventions with in-person discussions and debriefings to consolidate learning and enhance message retention (Boboc and Damaševičius, 2024; Bowman et al., 2020).

Furthermore, XR-based prevention initiatives should extend beyond adult–child abuse scenarios to address peer-perpetrated sexual victimization, a significant and under-recognized concern in adolescent populations (Finkelhor, 2025). Immersive prevention tools hold promise for targeting early sexualized behaviors and promoting prosocial, respectful peer interactions. Notably, emerging evidence from related fields suggests that XR interventions can effectively foster empathy, emotion recognition, and moral reasoning, particularly through role-exchange designs in VR, where participants alternate between victim and aggressor roles. These designs have been shown to produce superior outcomes in empathy development, moral reasoning, and behavioral intentions compared to single-role interventions (Boboc and Damaševičius, 2024; Gu et al., 2022; Seinfeld et al., 2018).

These findings collectively suggest that immersive role-playing and embodiment experiences may serve as powerful mechanisms for addressing empathy deficits, difficulties in recognizing harmful social scripts, and maladaptive behaviors among at-risk youth. Consequently, XR technology holds considerable potential as a novel modality for early intervention—not only for children vulnerable to CSA but also for those exhibiting problematic sexual behaviors or facing heightened risks of coercion or victimization within peer contexts. These mechanisms warrant further exploration in future XR-based CSA prevention designs, which should prioritize integrating empathic embodiment, role-exchange formats, and contextually sensitive content tailored to the developmental and cultural characteristics of the target population.

In contrast, therapeutic interventions using XR for children and adolescents who are victims of CSA remain notably underdeveloped. This review identified only preliminary efforts in this area (Endendijk et al., 2021). However, the study reviewed presents several limitations. It focused exclusively on therapists' perspectives, neglecting the experiences and views of child clients, which may differ substantially. Future research should incorporate age-appropriate assessments directly from children and adolescents to better capture their unique perspectives. Additionally, the study provided limited background information on both therapists and clients, precluding analysis of how clinical profiles or professional experience might influence the application and outcomes of the intervention. As an exploratory investigation, further research employing experimental and longitudinal designs is essential to rigorously evaluate the therapeutic effectiveness and underlying mechanisms of Vil Du?! in CSA treatment.

Despite these gaps, emerging evidence supports the potential of XR to complement trauma-focused cognitive-behavioral therapy (TF-CBT), particularly in treating post-traumatic stress disorder (PTSD) through immersive technologies (Eshuis et al., 2021). Although few studies have applied VR-enhanced TF-CBT specifically to CSA populations, related research in other trauma contexts has shown promising results, indicating a possible pathway for future applications. For example, studies involving adult populations demonstrate that virtual reality exposure therapy (VRET) can effectively reduce PTSD and depressive symptoms (Botella et al., 2017; Peskin et al., 2018; Reger et al., 2016) and support court preparation processes for survivors of sexual violence (Sigurvinsdottir et al., 2023). Similarly, experimental VR trauma paradigms have highlighted that emotion regulation flexibility and cognitive appraisal significantly influence post-traumatic symptom development, which may inform adaptations for interventions with children and adolescents (Schweizer et al., 2018).

Nonetheless, these findings should be interpreted with caution when applied to younger populations due to developmental differences and ethical considerations (Ligthart et al., 2021). Still, immersive VR holds promise as a tool to safely simulate distressing experiences in therapeutic contexts, particularly when integrated with evidence-based trauma models and adapted to developmental stages. There is a significant opportunity to explore the benefits of XR as an adjunct to conventional CSA therapies; however, this requires rigorous adherence to high ethical standards and robust technical safeguards to prevent re-victimization or iatrogenic effects.

## Limitations

This review presents several limitations, primarily stemming from the limited number of studies available in this emerging field. First, the small sample of studies reduces the generalizability of the findings, making it difficult to draw conclusions applicable to broader populations, settings, or cultural contexts. Additionally, the overall quality of the included studies and the lack of diversity in their samples and methodological designs increase the potential for bias. The considerable variability in study designs, intervention formats, and outcome measures further complicates direct comparisons and limits the synthesis of consistent results. Several studies relied on self-reported or short-term evaluations, making it difficult to determine long-term effects of XR interventions. This limitation is particularly evident in treatment studies, where evidence is still scarce and based mainly on therapists' perceptions rather than standardized outcome measures. As well, publication bias cannot be excluded, as most studies reported positive results, highlighting the need for more independent and controlled research in this emerging field.

Consequently, the applicability of the findings remains preliminary, and the reliability of the conclusions must be interpreted with caution. Future research should address these limitations by employing more rigorous, diverse, and methodologically consistent studies to strengthen the evidence base in this field.

## Conclusions

This review highlights that, while the use of XR technologies in CSA interventions remains in its early stages, it holds significant potential across three primary domains: professional training, prevention, and, to a more limited extent, therapeutic treatment. XR-based professional training has shown strong promise in improving practitioners' adherence to evidence-based protocols, enhancing interviewing skills, and fostering emotional preparedness in challenging investigative contexts. Prevention programs leveraging immersive and interactive XR tools have demonstrated effective engagement and skill-building in children and adolescents, particularly when grounded in evidence-based frameworks and supported by adult facilitation and in-person debriefing. Notably, prevention initiatives should broaden their scope to address peer-perpetrated sexual harm, capitalizing on VR's capacity to enhance empathy, moral reasoning, and prosocial behavior, as shown in related areas of youth violence prevention. In contrast, the therapeutic use of XR for CSA victims, particularly children and adolescents, remains largely exploratory. Current evidence does not suggest that XR can replace conventional psychological treatments, especially in such sensitive contexts, but it may become a valuable complementary tool. Future XR interventions in therapy must be grounded in trauma theory, rigorously evaluated through experimental and longitudinal designs, and carefully adapted to developmental stages. Prioritizing ethical safeguards, emotional safety, and cultural relevance is essential to prevent potential harm and ensure interventions are both effective and appropriate.

Consistent with these findings, it is recommended that immersive digital experiences be integrated with real-time, group-based interventions to consolidate learning, facilitate emotional processing, and enhance therapeutic outcomes. The quality and realism of immersive contexts also emerged as critical factors influencing efficacy. Future research should continue refining these interventions, developing AI-enhanced feedback systems, and assessing cost-efficiency and long-term effectiveness. By advancing these areas, XR technologies may become valuable adjuncts to traditional CSA interventions, offering innovative pathways to strengthen prevention, professional training, and therapeutic support for this highly vulnerable population.

## Data Availability

The datasets presented in this study can be found in online repositories. The names of the repository/repositories and accession number(s) can be found at: references.
